# Continuous Versus Routine EEG in Critically Ill Patients Without Primary Neurologic Condition: Analysis of a Randomized Controlled Trial

**DOI:** 10.1111/ene.70768

**Published:** 2026-09-28

**Authors:** Gregory Lepeu, Jan Novy, Vincent Alvarez, Kaspar A. Schindler, Stephan Rüegg, A. O. Rossetti

**Affiliations:** ^1^ Service de Neurologie, Department of Clinical Neurosciences Centre Hospitalier Universitaire Vaudois (CHUV) and Lausanne University Hospital Lausanne Switzerland; ^2^ Department of Neurology Hôpital du Valais Sion Switzerland; ^3^ Department of Neurology, Inselspital, Sleep‐Wake‐Epilepsy‐Center Bern University Hospital and University of Bern Bern Switzerland; ^4^ Department of Neurology University Hospital Basel and University of Basel Basel Switzerland

**Keywords:** continuous EEG, critically ill, prognosis, seizure burden

## Abstract

**Introduction:**

Continuous EEG (cEEG) is increasingly used in critically ill patients, driven by its superior seizure detection sensitivity over routine EEG (rEEG). However, cEEG is resource‐demanding, and whether its use translates into improved clinical outcomes in patients without primary neurologic conditions remains unknown.

**Methods:**

This is a post hoc analysis of the multicenter randomized CERTA trial, in which critically‐ill adults were randomized 1:1 to cEEG or two rEEG recordings. Six‐month mortality represented the primary outcome, while exploratory outcomes included functional outcome (Cerebral Performance Categories, CPC), detection of seizures/status epilepticus, ictal–interictal continuum features, medication changes, and infections within 4 weeks. Analysis was restricted to patients without primary neurologic injury.

**Results:**

Among 364 enrolled patients, 76 lacked a primary neurologic condition (cEEG: *n* = 34, rEEG: *n* = 42). Continuous EEG was not associated with mortality (*p* = 0.970) or functional outcome (CPC 1–2; *p* = 0.893). No seizures or status epilepticus were detected in either group. Rates of EEG periodic patterns, ictal–interictal continuum features, medication changes, and infections were comparable between groups.

**Conclusion:**

In critically ill adults without a primary neurologic condition, cEEG was not associated with measurable benefits in survival, neurological recovery, or epileptiform activity detection compared with repeated rEEG, in a cohort without detected electrographic seizures or status epilepticus. Pending larger, ideally prospective comparative studies, repeated routine EEG may represent a reasonable alternative in resource‐limited settings.

## 1 Introduction

Acute brain dysfunction, characterized by altered mental status and propensity to develop epileptiform activity, can arise in critically ill patients without primary neurologic conditions as a result of systemic homeostatic perturbation, inflammation, metabolic imbalance, or impaired cerebral perfusion [1]. Several studies have reported EEG abnormalities in this population, including periodic patterns and non‐convulsive seizures [2, 3, 4, 5, 6], which have been independently associated with poor outcomes. Non‐convulsive seizures and status epilepticus are of particular concern, as they are clinically indistinguishable from encephalopathy and have been associated with lasting brain injury [7, 8].

Continuous EEG (cEEG) is increasingly used in critically ill patients for its superior seizure detection sensitivity over routine EEG (rEEG) [9, 10, 11, 12], yet it is resource‐demanding [13] and its effect on clinical outcomes remains debated [9, 10, 14, 15]. This question has received particularly limited attention in patients without primary brain injury, where existing evidence relies exclusively on observational studies without comparison of EEG lengths [2, 3, 5, 6, 16, 17]. We attempted to fill this knowledge gap in an exploratory analysis of the CERTA trial [9], examining in critically ill patients without a primary neurologic condition whether cEEG is associated with better outcomes compared to repeated rEEG.

## 2 Methods

This is a post hoc analysis of prospectively collected data from the multicenter randomized CERTA trial (NCT03129438), which was ethically approved (project‐ID 2017–00268). Briefly, 364 critically ill adults with impaired consciousness and needing EEG for clinical purposes were randomized 1:1 to either cEEG (lasting 30–48 h) or two rEEG (each 20–30 min) across four Swiss hospitals. Patients receiving palliative care, undergoing invasive procedures within 48 h, or with recent seizures (within 36 h) and/or status epilepticus (within 96 h before randomization) were not enrolled. Methodological details have been published previously [9].

EEGs were visually analyzed by trained and certified epileptologists according to the American Clinical Neurophysiology Society (ACNS) standardized Critical Care EEG Terminology [18], including classification of seizures, status epilepticus, sporadic epileptiform discharges, and rhythmic or periodic patterns—generalized and lateralized periodic discharges (GPDs, LPDs), and generalized and lateralized rhythmic delta activity (GRDA, LRDA)—classified as qualifying or not for the ictal–interictal continuum (LPD, LRDA, GPD) according to ACNS guidelines [19].

The present analysis focuses on patients without a primary neurological condition, excluding those with stroke, intracranial hemorrhage, traumatic brain injury, CNS inflammation or infection, CNS neoplasm, recent brain surgery, or post‐anoxic encephalopathy. Clinical characteristics, neurological status before EEG, and post‐EEG outcomes were pre‐specified and prospectively collected, including pharmacological sedation and antiseizure medication (ASM) administration and modifications triggered by EEG results, and infections within 4 weeks. Mortality and functional outcome (Cerebral Performance Categories [CPC] 1–2 considered favorable) were assessed at 6 months by collaborators blinded to EEG allocation.

Statistical analyses were performed using Python 3.11 script and reported either as mean ± standard deviation (SD), or median and interquartile ranges (IQR), as appropriate. Differences across groups were assessed with *t*‐tests or *U*‐tests for continuous variables, and *χ* ^2^ tests or Fisher exact test for categorical variables. All tests were two‐sided (*p* < 0.05).

## 3 Results

Among the 364 critically ill adults enrolled in the CERTA trial, 76 without a primary neurological condition were included in this post hoc analysis (22 women (29%); mean age: 65.3 years (SD 12.8)). Etiologies included septic encephalopathy in 34 patients (45%), metabolic encephalopathy in 24 (32%), medication overdose in 5 (7%), alcohol or medication withdrawal in 3 (4%), and altered mental status of unknown cause in 13 (17%); categories were not mutually exclusive and 3 patients (3.9%) had more than one etiologic category. Median SAPS‐II score and Charlson Comorbidity Index scores on admission were respectively 45 (IQR 34–61) and 3 (1–4). Patients exhibited severe encephalopathy, with a median Glasgow Coma Scale (GCS) score of 4 (IQR 3–6) at randomization; 58 (76%) were mechanically ventilated and 39 (51%) were sedated with propofol and/or midazolam. EEG was primarily requested for suspected seizures (95%), defined as clinical events prompting EEG referral by the treating ICU team.

Patients were randomized to continuous EEG (cEEG; *n* = 34) or routine EEG (rEEG; *n* = 42). Table 1 illustrates clinically relevant variables stratified by EEG intervention. There were no significant differences between groups in demographic characteristics or severity of acute illness, as assessed by the Simplified Acute Physiology Score II (SAPS II).

**TABLE 1:** Patients' clinical characteristics and outcomes.

Variable	cEEG (*n* = 34)	rEEG (*n* = 42)	*p*	Test
Female gender (*n*, %)	12 (35)	10 (24)	0.399	Chi^2^
Age (mean, SD)	67.1 (10.3)	63.8 (14.5)	0.254	*t*‐test
mRs before admission (median, IQR)	2 (0–3)	2 (1–3)	0.835	*U*‐test
FOUR score on admission (median, IQR)	6 (4–8)	5 (4–8)	0.496	*t*‐test
GCS score on admission (median, IQR)	4 (3–7)	3 (3–6)	0.586	*U*‐test
SAPS II score (median, IQR)	45 (34–57)	47 (35–61)	0.952	*U*‐test
Charlson Comorbidity Index (median, IQR)	2 (1–3)	3 (1–5)	0.567	*U*‐test
Suspected seizures (*n*, %)	33 (97)	39 (93)	0.765	Chi^2^
Previous history of seizures (*n*, %)	3 (9)	4 (10)	1.000	Chi^2^
In Intensive Care Unit (*n*, %)	33 (97)	40 (95)	1.000	Chi^2^
Undergoing mechanical ventilation (*n*, %)	26 (77)	32 (76)	1.000	Chi^2^
Duration ventilation [h] (mean, SD)	236.4 (231.4)	181.2 (226.1)	0.253	*U*‐test
Received Midazolam (*n*, %)	12 (35)	15 (36)	0.949	Chi^2^
Midazolam dose at EEG start [mg/kg/h] (mean, SD)	0.083 (0.063)	0.134 (0.130)	0.574	*U*‐test
Received Propofol (*n*, %)	11 (32)	15 (36)	1.000	Chi^2^
Propofol dose at EEG start [mg/kg/h] (mean, SD)	0.694 (0.822)	1.287 (1.115)	0.132	*U*‐test
*Etiology*				
Septic encephalopathy (*n*, %)	15 (44)	19 (45)	1.000	Chi^2^
Metabolic encephalopathy (*n*, %)	10 (29)	14 (33)	0.906	Chi^2^
Alcohol/medication withdrawal (*n*, %)	1 (3)	2 (5)	1.000	Fisher
Medication overdose (*n*, %)	3 (9)	2 (5)	0.651	Fisher
Unknown (*n*, %)	6 (18)	7 (17)	1.000	Chi^2^
*EEG detections*				
Seizure/Status Epilepticus (*n*, %)	0 (0)	0 (0)	1.000	Fisher
Sporadic epileptiform discharges (n, %)	12 (35)	16 (38)	0.990	Chi^2^
Rhythmic or periodic pattern (n, %)	16 (47)	19 (45)	1.000	Chi^2^
GRDA (*n*, %)	10 (29)	11 (26)	0.957	Chi^2^
LRDA (*n*, %)	1 (3)	1 (2)	1.000	Fisher
GPD (*n*, %)	3 (9)	6 (14)	0.723	Fisher
LPD (*n*, %)	2 (6)	2 (5)	1.000	Fisher
Ictal‐interictal continuum (*n*, %)	6 (18)	9 (21)	0.902	Chi^2^
Sleep spindles (*n*, %)	17 (50)	8 (19)	**0.009**	Chi^2^
*Immediate outcome*				
Change in medication within 60 h (*n*, %)	14 (41)	14 (33)	0.642	Chi^2^
Subsequent EEG (*n*, %)	8 (24)	4 (10)	0.118	Fisher
Infections within 4 weeks (*n*, %)	5 (15)	9 (21)	0.480	Chi^2^
*Outcome at 6 month*				
Mortality (*n*, %)	14 (41)	16 (38)	0.970	Chi^2^
CPC (median, IQR)	2 (1–2)	2 (1–3)	0.629	*U*‐test
Good Outcome (CPC 1–2) at 6 month (*n*, %)	16 (47)	18 (43)	0.893	Chi^2^

*Note:* Bold values are significant.

Abbreviations: ASM, anti—seizure medication; cEEG, continuous electroencephalogram; CGS, Glasgow Coma Scale; CPC, Cerebral Performance Categories; FOUR, Full Outline of Unresponsiveness; GPD, generalized periodic discharges; GRDA, generalized rhythmic delta activity; IQR, interquartile range; LPD, lateralized periodic discharges; LRDA, lateralized rhythmic delta activity; NREM, Non‐Rapid Eye Movement; rEEG, routine EEG; SAPSII, Simplified Acute Physiology Score II; SD, standard deviation.

No seizures or status epilepticus were identified in either group (0/34 and 0/42 patients, respectively; 95% CI: 0%–10.3% and 0%–8.4%). Detection rates of rhythmic and periodic patterns (including features qualifying for the ictal–interictal continuum), and sporadic epileptiform discharges were similar between cEEG and rEEG. In contrast, sleep spindles were detected more frequently in the cEEG group (17/34 (50%) vs. 8/42 (19%), *p* = 0.009). Among other exploratory outcomes, rates of antiseizure medication changes following EEG were similar, as were durations of mechanical ventilation and infections. Finally, there was no difference in 6‐month mortality (primary outcome: 14/34 (41%) vs. 16/42 (38%), absolute risk difference 3.0%; 95% CI: −19.4% to 25.4%, *p* = 0.970, Table 1), nor proportion of favorable neurological outcome (CPC 1–2).

## 4 Discussion

In this post hoc analysis of the CERTA trial, cEEG was not associated with measurable benefits over repeated rEEG in critically ill patients without a primary neurologic condition, either in terms of long‐term mortality, functional outcome, or detection of epileptiform activity.

Existing studies on EEG in non‐neurological critical illness have used either continuous monitoring [2, 3, 5, 6] or short recordings [16, 17], but none have directly compared the two strategies. To our knowledge, this represents the first comparison of EEG recording lengths in this etiological group, originating from a randomized cohort. The lack of prognostic advantage associated with cEEG is consistent with findings from the whole CERTA cohort [9], and subgroup analyses in traumatic brain injury [20] and cardiac arrest [21], suggesting a pattern that holds regardless of the underlying etiology.

Non‐convulsive seizures and status epilepticus were absent in the present cohort, consistent with recent studies in septic and metabolic encephalopathy [5, 6], but lower rates than reported in other cohorts of critically ill patients without primary neurologic injury, where seizure prevalence ranged from 10% to 16% [2, 3, 4, 17]. This discrepancy likely reflects differences in patient selection, particularly regarding the handling of patients with prior clinical seizures. In the present study, patients with recent seizures (within 36 h) and/or status epilepticus (within 96 h before randomization) were excluded. Previous clinical seizures occurrence has been consistently identified as a key predictor of subsequent seizure activity in analyses reporting higher seizure prevalence [2, 3, 4, 17]. These selection differences likely contributed to the very low seizure yield observed in our cohort. This absence is particularly relevant, as the principal mechanistic rationale for cEEG superiority over rEEG is improved seizure detection, which could not be operationalized in this cohort. However, even in the full CERTA trial, where cEEG detected substantially more seizures and led to more treatment modifications, no outcome benefit was observed [9].

Beyond seizure detection, rhythmic and periodic patterns were a frequent finding in our cohort, consistent with prior reports in non‐neurological critical illness [2, 4, 5, 6]. This is clinically relevant, as these EEG features have been associated with poor outcomes, delirium severity, and sepsis‐associated encephalopathy [5, 6, 17, 22, 23, 24]. As opposed to the whole CERTA cohort [9], cEEG did not detect these patterns more frequently than rEEG. Sleep spindles were observed significantly more often with cEEG, likely reflecting increased opportunity for sleep sampling during longer recordings. Sleep spindles are of interest because they have previously been associated with favorable outcomes [24]: this suggests that cEEG provides more prognostic information than rEEG [24, 25].

This study should be interpreted with limitations. The post hoc design precludes causal inference. The exclusion of patients with recent ictal events restricts generalizability. Sedation in half of the cohort may also have attenuated epileptiform activity in both arms, but this is a common occurrence in critically‐ill patients. In addition, detailed information on the exact clinical semiology prompting EEG referral (e.g., motor phenomena, eye deviation or unexplained altered consciousness) was not collected in the parent trial. These constraints notwithstanding, the randomized EEG allocation, EEG interpretation adhering to recognized guidelines [18], blinded outcome assessment, and multicenter recruitment represent in our view relevant aspects to enhance the internal validity of the findings and orient future studies on the topic. Despite the absence of statistically significant differences or consistent trends across clinically relevant endpoints, and the relatively modest sample size, clinically relevant effects cannot be formally excluded.

In conclusion, pending larger, ideally prospective comparative studies, repeated rEEG may represent, particularly in resource‐limited settings, a reasonable approach in critically ill adults without primary neurologic injury or recent clinical seizures/status epilepticus and undergoing EEG for unexplained altered mental status since seizures appear to be very uncommon in this population.

## Author Contributions

**Vincent Alvarez:** data curation, writing – review and editing. **A. O. Rossetti:** conceptualization, funding acquisition, writing – original draft, data curation, project administration, supervision. **Stephan Rüegg:** data curation, writing – review and editing. **Jan Novy:** data curation, writing – review and editing. **Kaspar A. Schindler:** data curation, writing – review and editing. **Gregory Lepeu:** conceptualization, writing – original draft, investigation, formal analysis.

## Conflicts of Interest

The authors declare no conflicts of interest.

## Data Availability

The data that support the findings of this study are available from the corresponding author upon reasonable request.
